# Insights into Photothermally Enhanced Photocatalytic U(VI) Extraction by a Step-Scheme Heterojunction

**DOI:** 10.34133/2022/9790320

**Published:** 2022-10-11

**Authors:** Yifeng Zhang, Haorong Sun, Feixue Gao, Shuo Zhang, Qingzhi Han, Jing Li, Ming Fang, Yawen Cai, Baowei Hu, Xiaoli Tan, Xiangke Wang

**Affiliations:** ^1^MOE Key Laboratory of Resources and Environmental System Optimization, College of Environmental Science and Engineering, North China Electric Power University, Beijing 102206, China; ^2^Key Laboratory of Theoretical and Computational Photochemistry, Ministry of Education, College of Chemistry, Beijing Normal University, Beijing 100875, China; ^3^Key Laboratory of Photochemical Conversion and Optoelectronic Materials, Technical Institute of Physics and Chemistry, Chinese Academy of Sciences, Beijing 100190, China; ^4^School of Life Science, Shaoxing University, Shaoxing 312000, China

## Abstract

In this work, a CdS/BiVO_4_ step-scheme (S-scheme) heterojunction with self-photothermally enhanced photocatalytic effect was synthesized and applied for efficient U(VI) photoextraction. Characterizations such as transient absorption spectroscopy and Tafel test together confirmed the formation of S-scheme heterojunctions, which allows CdS/BiVO_4_ to avoid photocorrosion while retaining the strong reducing capacity of CdS and the oxidizing capacity of BiVO_4_. Experimental results such as radical quenching experiments and electron spin resonance show that U(VI) is rapidly oxidized by photoholes/^•^OH to insoluble UO_2_(OH)_2_ after being reduced to U(IV) by photoelectrons/^•^O_2_^−^, which precisely avoids the depletion of electron sacrificial agents. The rapid recombination of electron-hole pairs triggered by the S-scheme heterojunction is found to release large amounts of heat and accelerate the photocatalysis. This work offers a new enhanced strategy for photocatalytic uranium extraction and presents a direction for the design and development of new photocatalysts.

## 1. Introduction

Nuclear energy is receiving more and more attention and development because of its high energy density, low carbon, etc. [1]. Uranium, as the main material used in the nuclear industry, is entrained in the wastewater and released in large quantities (mainly in the form of soluble UO_2_^2+^) during the mining and utilization process, causing great pollution to the environment. [2] Most importantly, U(VI) is highly susceptible to mixing into drinking water, entering organisms, and eventually accumulating in the human body along the food chains. Its radioactivity and heavy metal toxicity can induce a variety of fatal diseases and even death. [3] Therefore, it is an urgent need to efficiently extract uranium from water bodies, which would address both the shortage of uranium resource and environmental pollution. Researchers have also been trying to find strategies for the efficient extraction of uranium from water bodies [4–7].

Photocatalytic extraction has attracted great attention because it can directly use solar energy to degrade/remove pollutants without additional energy supply and secondary pollution. [8–10] However, many single photosensitive semiconductors have suboptimal photo-oxidation/reduction performance for contaminants due to various reasons, including inappropriate band gaps, high photogenerated charge recombination rates, and susceptibility to photocorrosion. What's more, the reduction/oxidation ability heavily relies on the potentials of the conduction band minimum (CBM) and valence band maximum (VBM) of a photocatalyst. For example, to reduce U(VI) efficiently, the CBM of the photocatalyst needs to be negative to the reduction potential of U(VI)/U(IV) (0.411 V) to a certain extent (sufficient overpotential). CdS is an n-type semiconductor with a strong light capture capability and a strong reducing ability (E_VBM_≈−0.75 V vs. NHE), which is widely used in water splitting andorganic matter degradation [11–13]. However, the photocorrosion of CdS has hindered its large-scale application. Most current approaches to prevent photocorrosion focus on the rapid elimination of photoholes in CdS, such as adding a hole-trapping agent to the catalytic system or constructing a heterojunction to facilitate the transfer of the photoholes [14, 15]. The former increases the cost of using CdS and leads to lowering the possibility of real application, while the latter usually sacrifices its strong oxidation ability. BiVO_4_ as a bismuth-based semiconductor has excellent light absorption properties due to the valence band consisting of Bi 6 s and O 2p hybridized orbitals, resulting in a reduced band gap width, and its low VBM position gives it strong photocatalytic oxidation performance, thus it is widely used in photocatalysis [16].

Very recently, the discovery of step-scheme (S-scheme) heterojunctions provides an efficient solution to the above problems, for example, the production of H_2_ and H_2_O_2_ could be significantly increased by inhibiting the photocorrosion of CdS by constructing the S-scheme heterojunctions by Tang and Mirsadeghi, respectively. [17, 18] In simple terms, an S-scheme heterojunction is a special type of Z-scheme heterojunction, but with more severe conditions. [19] It is well known that the Z-scheme heterojunction is usually composed of a reduction-type photocatalyst (RP) and an oxidation-type photocatalyst (OP) matching with a stepped energy band structure. In detail, the CBM and VBM of RP are more negative than those of OP, and the VBM of RP is more positive than the CBM of OP. However, for S-scheme heterojunctions, in addition to meeting the above conditions, the positions of the Fermi energy levels of RP and OP are also required, i.e., the position of the Fermi energy level of RP should be higher than that of OP (in other words, the work function of RP is lower than that of OP). In this way, without light irradiation, driven by the potential difference of the Fermi levels, the electrons in RP keep diffusing to OP to form an electric field directed from RP to OP, leaving holes in RP. [20] Once the light is applied, the photoelectrons in the conduction band (CB) of OP could be rapidly transferred to the valence band (VB) of RP to recombine with holes under the action of the built-in electric field. As a result, the photoelectrons in the CB of RP with strong reduction potential over the CB of OP and the holes with strong oxidation potential over the VB of RP could be successfully retained. In this way, the electron-hole complexation occurring inside the component is reduced and a reduction-oxidation potential superior to that of the conventional type II heterojunction is obtained. In addition, it has been reported that the recombination of photogenerated electron-hole pairs releases energy outward in the form of heat [21]. The released heat usually causes an elevation of the local temperature, which in turn promotes charge separation and accelerates the photocatalytic reaction. [22, 23] However, the photothermal effect in S-scheme heterojunctions is seldom reported.

Inspired by the aforementioned advantages of S-scheme heterojunction, in this work, an S-scheme heterojunction based on BiVO_4_ and CdS is constructed to achieve efficient photocatalytic U(VI) extraction from water. The S-scheme heterojunction brings two advantages: firstly, thanks to the driving force generated by the built-in electric field, the photoelectrons of BiVO_4_ are rapidly transferred to CdS for fast recombining with holes, accompanied by a large amount of heat release. This thermal effect was found to increase the local temperature and greatly enhance the catalytic activity of the CdS/BiVO_4_ and accelerate the photoextraction process of U(VI); secondly, the S-scheme heterojunction enables the photocatalyst to inhibit the photocorrosion of CdS while retaining the strong reducing ability of CdS and the strong oxidizing ability of BiVO_4_, which allows uranium to be rapidly oxidized to U(VI) again after being reduced to U(IV). The present work demonstrates a new strategy for efficient photocatalytic U(VI) extraction by an S-scheme heterojunction, which could be accelerated by a self-photothermal effect.

## 2. Results and Discussions

### 2.1. Materials and Characterizations

**Figure 1:**
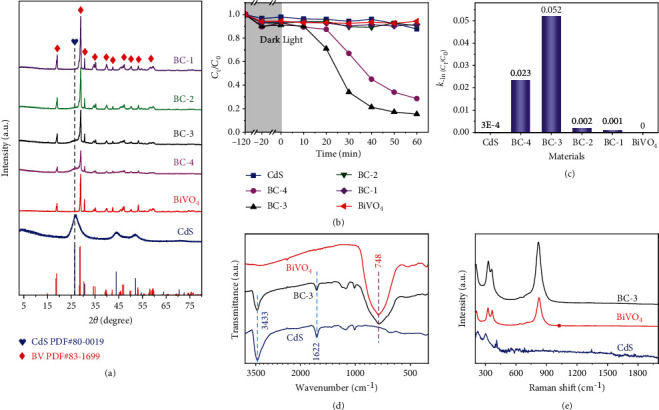
XRD patterns of as-synthesized samples (a); Photocatalytic experiment of U(VI) with different materials (*C*_U(VI) initial_ = 70 ppm, *T* = 25°C, pH 5.0) (b) and the corresponding *k* value of the first-order kinetic fitting (c); FTIR spectra (d) and Raman spectra (e) of the as-synthesized materials.

X-ray diffraction spectra are firstly used to investigate the crystal structures of the obtained samples.  Figure 1(a) shows that the synthesized BiVO_4_ belongs to the monoclinic system (JCPDS card No. 83-1699). As for CdS, its characteristic peaks are found at 26.55°, 44.04°, and, 52.16°, corresponding to the (111), (220), and (311) crystal planes of the cubic CdS (JCPDS card No. 80-0019), respectively. For the composites of BC-n, all the diffraction peaks could be well indexed to BiVO_4_ and CdS, indicating a successful combination. Given the importance of uranium, U(VI) is used as a target to test the photocatalytic performance of six samples. As can be seen from Figure 1(b) U(VI) adsorption-desorption equilibrium could be reached for all the six as-synthesized samples in 2 h in dark. The adsorbed capacities of the six samples for U(VI) are very weak (Figure S1), which may be because it is difficult to form a chemical bond with U(VI) and only a small amount of U(VI) can be absorbed by the negatively charged surface (Figure S2). It should be noted that the removal abilities of CdS, BC-2, BC-1, and BiVO_4_ for U(VI) are still not obvious even as the light is turned on, while BC-3 and BC-4 exhibit a superior removal ability in 60 min (Figure 1(b)). Especially for the BC-3, over 85% of U(VI) is removed under visible light irradiation after 1 h. However, the U(VI) concentration decreased slowly in the first 10 min of light irradiation and then decreased rapidly (the black line in Figure 1(b)), showing an inverted “S” shaped curve. This phenomenon has also been observed in our previous work and some other works, [24, 25] which is different from the normal photocatalytic experiments that usually reach the maximum reaction rate at the beginning of light irradiation and then gradually decrease with prolonging the catalytic time. [2, 26] The inverted “S” shaped curve usually corresponds complicated reaction mechanism, which will be carefully investigated later. To observe the photocatalytic ability, a pseudo-first-order model is utilized to simulate the photocatalytic process by plotting the -ln(*C*_t_/*C*_0_) vs. time curve (*C*_0_: 70 ppm, the initial U(VI) concentration; *C*_t_: U(VI) concentration at time *t* (min)) (Figure S3) and the plots of first-order rate constant (*k*) is calculated and presented in Figure 1(c). There is no doubt that the BC-3 has the highest rate constant of 0.052 min^−1^. Based on the above results, BC-3 is selected to perform the subsequent experiments. FTIR spectra are used to give an understanding of the vibration modes of BC-3. By comparing the FTIR spectra of BC-3, BiVO_4,_ and CdS (Figure 1(d)), all the characteristic absorption bands of BiVO_4_ and CdS are found in the FTIR spectrum of BC-3. An obvious absorption band at 748 cm^−1^ is ascribed to the antisymmetric stretching vibration of the VO_4_ group, and the absorption bands of O-H corresponding to the adsorbed water molecules on BC-3 are found at 3433 and 1622 cm^−1^. [27, 28] However, the stretching vibration absorption band of the S-Cd bond at 659 cm^−1^ in the infrared spectrum of BC-3 is weak and cannot be discriminated because it is obscured in the absorption band at 748 cm^−1^ of BiVO_4_. [29] The Raman spectrum of BiVO_4_ (Figure 1(e)) shows four peaks at 212, 326, 365, and 830 cm^−1^, and the peaks of BC-3 are almost identical to that of BiVO_4_, which also has four peaks. However, the two peaks at around 300 and 410 cm^−1^ of CdS are too weak to be observed in the Raman spectrum of BC-3. Thermogravimetry curve (Figure S4) shows that BC-3 has good thermal stability in the temperature range of 20~500°C. Figure S5 shows a type IV N_2_ ad-desorption isotherm of BC-3, and the BET surface area is evaluated to be 76.88 m^2^/g. The pore size distribution curve (the inset in Figure S5) shows there are lots of mesopores.

The morphology of the as-synthesized samples is investigated by FESEM. Figure S6 shows that CdS is in the form of small nanoparticles, while BiVO_4_ exhibits a surface roughed rod-like microstructure (Figure S7) with a length of 6 ~ 10 *μ*m and a width of ~0.8 *μ*m. The rough surface of BiVO_4_ makes it easy to adhere to CdS nanoparticles and facilitate photocatalysis, yet it also makes it difficult to distinguish the attached CdS nanoparticles at the same time (Figure S8A). However, in the TEM images of BC-3 (Figure S8B), it can be seen that there are a large number of small particles attached to the edge of the rod. What is more, the 0.33 nm crystal plane spacing attributed to CdS was observed in the HRTEM image (Figure S8C). Therefore, it can be proved that the small CdS particles are attached to the BiVO_4_ micron rods. Moreover, the SAED pattern (the inset in Figure S8B) also confirms the presence of polycrystalline CdS (bright white circle) and single-crystal BiVO_4_ (bright dots). The energy dispersive spectra (EDS) mappings are utilized to investigate the elemental distribution. From Figure S8D–I, five elements of O, V, Bi, S, and Cd can be observed, and all the elements exhibit a uniform distribution. After photocatalysis, the morphology of Half-used BC-3 (BC-3 for photocatalysis for 10 min) does not change much compared to BC-3 (Figure S9A and B). However, the surface of Used-BC-3 was found to be covered by a large number of nanosheets (Figure S9C). Moreover, by comparing the EDS mapping of Used BC-3 (Figure S10, Used BC-3 refers to BC-3 for photocatalysis for 60 min) and BC-3 (Figure S8D–I), it was found that the surface of Used BC-3 contains a large number of uranium elements, which confirms the successful extraction of U(VI) by BC-3.

### 2.2. Discussion of Photocatalysis Mechanism

**Figure 2:**
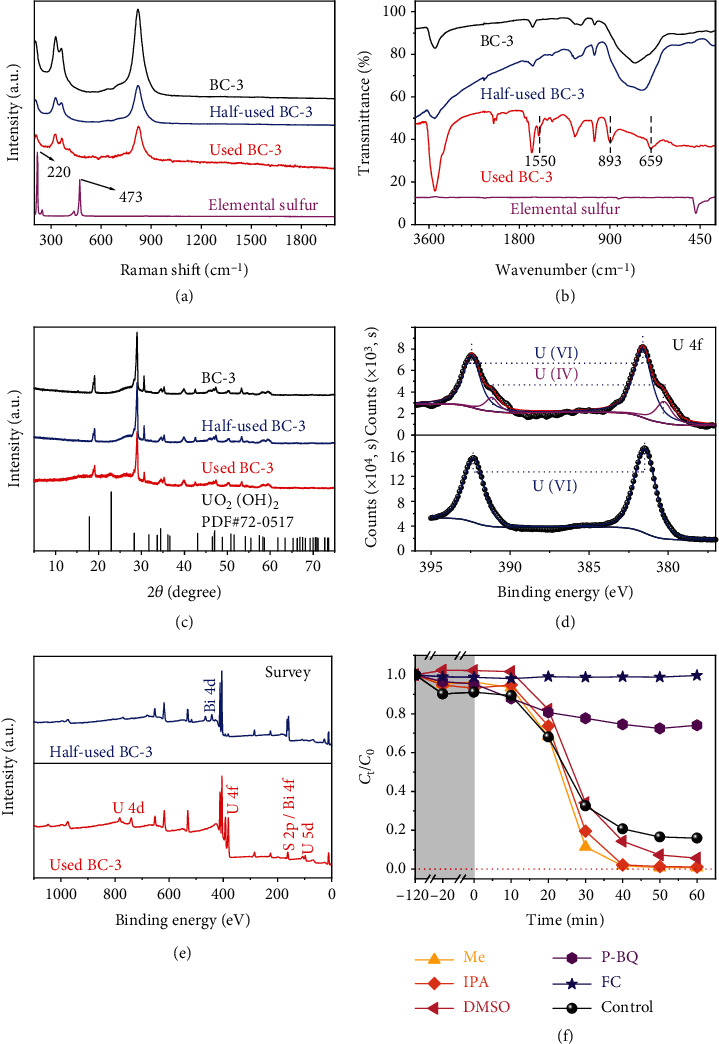
Raman spectra (a), FTIR spectra (b), and XRD patterns (c) of BC-3 at different stages in the photocatalytic reaction. Comparison of the high-resolution XPS spectrum of U 4f (d) and XPS survey spectra (e) in Half-used BC-3 and Used BC-3. The photocatalytic experiments with adding different trapping agents (f).

It is well known that CdS is prone to photocorrosion when it is used as a photocatalyst, resulting in the formation of a zero-valent S element with the release of Cd^2+^. [30–32] However, in this work, no characteristic peaks of zero-valent sulfur are founded in Raman spectra of the Half-used/Used BC-3 (Figure 2(a)), indicating that CdS does not undergo photocorrosion during the photocatalytic process. In addition, the ICP-MS shows that the concentration of Cd^2+^ in the separatd filtrate is 2.764 mg/L, which was tens of times less than the 68.086 mg/L reported in the work where photocorrosion occurred, [33] further confirming that photocorrosion was inhibited. In the FTIR spectra (Figure 2(b)), the spectrum of Half-used BC-3 is almost unchanged compared to that of BC-3. But the absorption band corresponding to O-H at 3436 cm^−1^ for Used BC-3 is stronger than that for BC-3, and a new absorption band corresponding to the U-O bond is also found at 893 cm^−1^, which implies an increase in the amount of O-H in Used BC-3 and the incorporation of uranium. [34] The small and sharp absorption band at 1550 cm^−1^ should be assigned to the N=O stretching mode of NO_3_^−^, which derives from the uranium source-UO_2_ (NO_3_)_2_·6H_2_O. [35] As for the XRD patterns (Figure 2(c)), no obvious difference is observed between BC-3 and Half-used BC-3. However, there is a new diffraction peak that appeared at 22.9° in the XRD pattern of the Used BC-3, the peak is classified as the diffraction peak on the (111) crystal plane in uranyl hydroxide (UO_2_(OH)_2_, JCPDS card No. 72-0517). To demonstrate the photocatalytic process, XPS characterizations on the samples at different reaction stages are performed. As shown in Figure 2(d), the characteristic peaks at 380.3 and 391.1 eV demonstrate the presence of U(IV) in Half-used BC-3, [33] suggesting that the reduction of U(VI) occurred (The survey spectra and high-resolution spectra are shown in Figure 2(e), S11, and S12, respectively.). However, for the Used BC-3, although the U(IV) could still be observed, the ratio of it decreased greatly (according to the ratio of the area of the subpeaks), and almost could not be observed in a repeated experiment (Figure S13). It should be noted that the Half-used BC-3 was taken after 10 min reaction corresponding to the slow reaction process, and the Used BC-3 was taken after 60 min reaction corresponding to the fast reaction process. Considering the decrease tendency of the photocatalytic curve, the decrease/dismiss of U(IV) means the reoxidization to solid U(VI).

**Figure 3:**
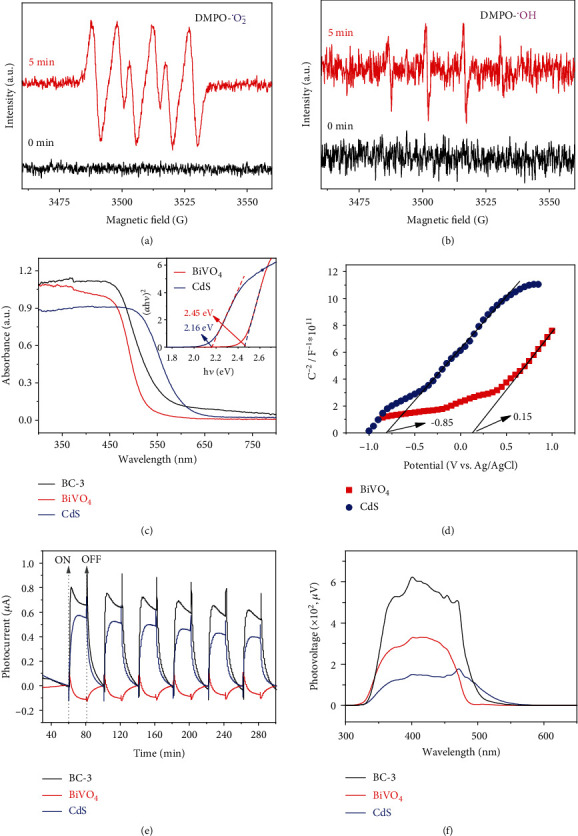
DMPO spin-trapping ESR spectra with 0.5 mg/mL BC-3 in the dark and under irradiation for 5 min at room temperature (the light conditions are the same as the photocatalytic experiments): DMPO-OH (a) and DMPO-^•^O_2_^−^ (b). UV-Vis DRS (the inset is the corresponding (*α*h*ν*)^2^ vs. photon energy curves of BiVO_4_ and CdS (c), Mott-Schottky plots (d), Transient photocurrent response (in 70 ppm U(VI), 0 V bias vs. Ag/AgCl) (e), SPV signal (f) of the as-synthesized samples.

To figure out the deep mechanism of the transform of U(VI) (l) → U(IV) (s) → U(VI) (s), quenching experiments are performed. As can be seen in Figure 2(f), the addition of FC (for e^−^ capture) and P-BQ (for ^•^O_2_^−^ capture) inhibits the photocatalytic reactions, while Me (for *h*^+^ capture), IPA (for ^•^OH capture), and DMSO (for ^•^OH capture) accelerate the catalytic rate to varying degrees. The ability of FC to capture photoelectrons is stronger than that of U(VI), so the existence of FC completely inhibits the reduction of U(VI). Me is an ideal hole trapping agent, and its addition enhances the utilization of photogenerated electrons. For ^•^O_2_^−^, it is usually formed from the combination of electrons and dissolved O_2_ in water (O_2_ + e^−^ → ^•^O_2_^−^), [36] The result that ^•^O_2_^−^ trapping inhibits U(VI) removal proves that it plays a positive role in U(VI) reduction. The mechanism of the ^•^OH radical trapping agent promoting the reaction is inferred from the following two aspects: on the one hand, the removal of ^•^OH creates a favorable environment for the reduction reaction; on the other hand, ^•^OH comes from the combination of water and photoholes (H_2_O + *h*^+^ → ^•^OH + H^+^), so the decrease of ^•^OH promotes more photoholes to oxidize water, thus reducing the recombination rate of photogenerated charges and improving the utilization rate of the photoelectrons. [37] In addition, the ESR spin-trap technique was implemented to more visually confirm the generation of ^•^O_2_^−^ and ^•^OH in the photocatalytic process. As shown in Figures 3(a) and 3(b), under dark conditions, the signals of DMPO-^•^O_2_^−^ and DMPO-^•^OH could not be detected, but after light irradiation for 5 min, they are both detected. The high-intensity DMPO-^•^O_2_^−^ signal implies a large production of ^•^O_2_^−^, which is very favorable for the reduction of U(VI). The weak DMPO-^•^OH signal implies less ^•^OH production, but the quantification of ^•^OH by fluorescence method (Figure S14A and B) revealed that its amount increased rapidly with the increase of irradiation time. Based on the above evidence, the photocatalytic process is reasonably inferred as follows: a large number of photoelectrons and photoholes are generated by BC-3 under light irradiation (Eq. (1)), and then the photoelectrons could reduce U(VI) to U(IV) (Eq. (2)) or generate ^•^O_2_^−^ with dissolved oxygen which could also reduce U(VI) (Eq. (3) and (4)). At the same time, partial photoholes directly oxidize the reduced U(IV) to insoluble U(VI) (in the form of UO_2_(OH)_2_); the remaining photoholes oxidize U(IV) through the ^•^OH generated by their reaction with water (Eq. (5) and (6)). [38] Also, the reduction may dominate in the slow reaction stage, and the reoxidization dominates in the fast reaction stage.

(1) $$\begin{array}{c} \text{BC}-3+hv⟶{e_{\text{BC}-3}}^{-}+{h_{\text{BC}-3}}^{+}, \end{array}$$

(2) $$\begin{array}{c} \text{U}\left(\text{VI}\right)+{e_{\text{BC}-3}}^{-}⟶\text{U}\left(\text{IV}\right), \end{array}$$

(3) $$\begin{array}{c} {\text{O}}_{2}+{\text{e}}^{-}{⟶}^{\cdot }{{\text{O}}_{2}}^{-}, \end{array}$$

(4) $$\begin{array}{c} {}^{\cdot }{{\text{O}}_{2}}^{-}+\text{U}\left(\text{VI}\right)⟶\text{U}\left(\text{IV}\right)+{\text{O}}_{2}, \end{array}$$

(5) $$\begin{array}{c} {\text{H}}_{2}\text{O}+{h_{\text{BC}-3}}^{+}⟶{\text{H}}^{+}{+}^{\cdot }\text{OH}, \end{array}$$

(6) $$\begin{array}{c} \text{U}\left(\text{IV}\right){+}^{\cdot }\text{OH}/h^{+}⟶\text{U}\left(\text{VI}\right). \end{array}$$

The generation and transfer mechanism of photogenerated charges play a crucial role in the photocatalytic process, therefore, their optoelectronic properties are intensively studied. The UV-Vis DRS spectra (Figure 3(c)) show that the absorption edges of BiVO_4_ and CdS are at approximately 530 nm and 580 nm, respectively, and~560 nm for BC-3. The inset in Figure 3(c) is the data calculated using the Kubelka-Munk formula, indicating the forbidden bandwidths (E_g_) of CdS and BiVO_4_ are 2.16 eV (~574 nm) and 2.38 eV (~520 nm), respectively. [39]. In the Mott-Schottky (M-S) test, the slopes of the M-S curves of BiVO_4_ and CdS (Figure 3(d)) are both positive, which means that they both are n-type semiconductors. In addition, the data show that the flat band potential (*E*_fb_) of BiVO_4_ and CdS are 0.15 V and -0.85 V (vs. Ag/AgCl), respectively. Thus, the CB of BiVO_4_ and CdS are estimated to be 0.25 V and -0.75 V (vs. NHE at pH 7), and the valence band (VB) potentials of BiVO_4_ and CdS are calculated to be 2.63 V and 1.41 V, respectively. [33] This means that CdS and BiVO_4_ form a type II heterojunction (Figure S15A), in which the CB of CdS is higher than the reduction potential of U(VI)/U(IV) (0.41 V) by 1.16 V, while the CB of BiVO_4_ is only higher than that by 0.15 V. In general, a sufficiently large overpotential is necessary to ensure the generation of chemical reactions. [40, 41] The small overpotential of BiVO_4_ could not lead to the photoreduction of U(VI), as evidenced by the results of the photocatalytic experiments in this work. Therefore, the photocatalytic mechanism could not be explained by the conventional photoelectron transfer path in a type II heterojunction, because the photoelectrons of CdS transferred to the CB of BiVO_4_ cannot initiate the photocatalytic reaction. The fact that photocatalysis of pure CdS to U(VI) is poor even if the overpotential is enough, indicates the photogenerated electrons suffer a serious recombination rate with photoholes. However, after complexation with BiVO_4_, the photocatalysis is greatly enhanced, indicating the recombination of photoelectrons with photoholes becomes less, or in other words, the photoholes generated in CdS are consumed by BiVO_4_. Such electron/hole transfer route has been uncovered in some other systems recently and named Z-scheme heterojunction. [42] This could be indirectly proved by the anodic photocurrent of CdS and the cathodic photocurrent of BiVO_4_ (Figure 3(e), see the SI for more). [43, 44] Also, from Figure 3(e), BC-3 exhibits its excellent charge separation efficiency due to the strongest photocurrent response, which may thus induce superior optoelectronic performance, as demonstrated by the surface photovoltage (SPV) measurements (Figure 3(f)) where the strongest SPV signal shown by BC-3 implying the highest charge separation rate. [45] In the EIS spectrum (Figure S16), the smallest radius exhibited by BC-3 indicates that it has the smallest charge transfer resistance among the three samples. [46] In the Z-scheme configuration (Figure S15B), the photoelectrons of BiVO_4_ recombine with the photoholes of CdS retaining the strongly reducing electrons in the CB of CdS and the strongly oxidizing holes in the VB of BiVO_4_. This could be intuitively confirmed by the time-resolved fluorescence and TA spectra.

**Figure 4:**
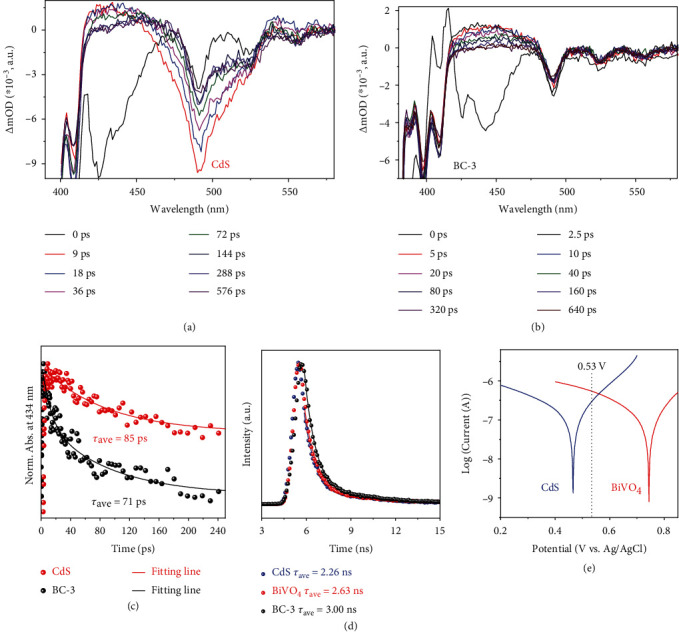
TA spectra of CdS (a) and BC-3 (b). Time profiles of normalized transient absorption at 434 nm (c), time-resolved fluorescence (d), and Tafel curves (e) of the as-synthesized samples.

As shown in Figure 4(a) and 4(b), the transient absorption (TA) spectra were obtained under the excitation of 375 nm light for CdS and BC-3. The absorption in the visible light region (400-700 nm) in the TA spectra is usually attributed to the surface-trapped holes. [47, 48] As the trapped holes on the surface are transferred or extinguished, the absorption intensity at the corresponding position in the transient absorption spectrum will gradually weaken until the absorption intensity is 0. Therefore, the lifetime of the cavity can be obtained by analyzing the variation of the absorption intensity with time at specific locations in the visible light region of the TA spectrum. [49] Thus, the time profiles of TA spectra probed at 434 nm were fitted with a biexponential function (Figure 4(c)) to further evaluate the decay kinetics of photoholes. The average lifetime of surface-trapped holes of CdS is shortened from 85 ps to 71 ps after loading onto BiVO_4_, indicating that the holes in CdS are rapidly trapped by electrons from BiVO_4_. This result solidly validates the Z-scheme transfer of electrons from BiVO_4_ to CdS. Also, from the time-resolved fluorescence (Figure 4(d), See SI for relevant fitting data), the electron proportion for the radiative process of BC-3 is the lowest (59.88%), which represents the recombination of photogenerated electron-hole pairs in BC-3 is greatly suppressed. [20] Correspondingly, the electron proportion (40.12%) with a long lifetime (*τ*_2_, corresponding to the non-radiation process) of BC-3 is significantly higher than those of CdS and BiVO_4_, implying that large quantities of photoelectrons undergo the nonradiation process.

**Figure 5:**
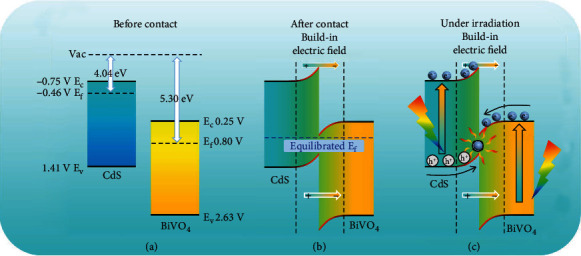
The intrinsic energy level distribution of CdS and BiVO_4_ (a). The energy level change after the contact between CdS and BiVO_4_ (b). The S-scheme transfer mechanism of photogenerated carriers in BC-3 under illumination.

The Tafel curves are recorded to discern the flow direction of charges at the interface of CdS and BiVO_4_. As shown in Figure 4(e), the redox equilibrium potential of CdS is more negative compared to that of BiVO_4_, and the open-circuit voltage of CdS/BiVO4 (0.53 V vs. Ag/AgCl) lies between the redox equilibrium potentials of CdS and BiVO_4_ from the Tafel results. Based on Liu's results, [50] it can be inferred that once CdS and BiVO_4_ come into contact, electrons will flow from CdS to BiVO_4_, i.e., the Fermi level (E_f_) of CdS is higher than that of BiVO_4_. Moreover, our test results are also supported by previous reports that the work functions of CdS and BiVO_4_ are measured to be 4.04 eV and 5.3 eV, respectively [51, 52]. Such charge flow has been called an S-scheme in the context of the Z-scheme by Yu in 2019 [53]. Consequently, the charges transfer path could be proposed as demonstrated in Figure 5. Before contact, BiVO_4_ and CdS each maintain their intrinsic energy level scheme (Figure 5(a)). When the two semiconductors are in close contact (Figure 5(b)), the electrons in CdS are spontaneously transferred to BiVO_4_ through the interface, given that the Fermi energy of CdS is higher than that of BiVO_4_, resulting in an upward bending for CdS and downward bending for BiVO_4_ of the energy band. Most importantly, an electric field was built directed from CdS to BiVO_4_. After light irradiation (Figure 5(c)), both CdS and BiVO_4_ could generate photoelectrons and photoholes. However, driven by the built-in electric field and benifit from the band bending at the interface, the photoelectrons in the CB of BiVO_4_ could combine with the holes in the VB of CdS. In such manner, the photoelectrons in CdS and the photoholes in BiVO_4_ were maintained, with the strong reducing and oxidizing abilities.

**Figure 6:**
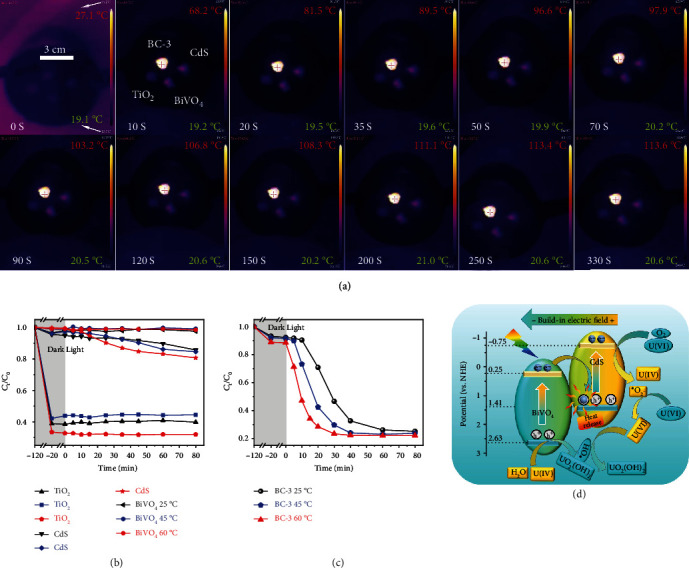
The infrared photothermal images of the four samples exposed to xenon lamp (350 W, >420 nm) at different times. The samples (*m* = 0.2 g) are placed on a constant temperature bench (exposed to air) for testing with commercial TiO_2_ as a reference. Photocatalytic activity of BC-3 (b) and CdS, BiVO_4_, TiO_2_ (c) for U(VI) at different temperatures. The mechanism of photocatalytic extraction of U(VI) by BC-3 (D).

It should also be noted that there is strong recombination of photoelectrons in BiVO_4_ and the photoholes in CdS, while the fluorescence intensity of BC-3 is the lowest among the three samples (Figure S17), therefore, the energy released by the recombination should be the other form than light, for example, heat, i.e. photothermal effect. [21, 54] The released heat usually leads to an increase in the local temperature of the catalyst, which promotes charge separation and accelerates the photocatalytic reaction. [22, 23, 55] To indicate the photothermal effect, the infrared image of the samples under the light irradiation was shown in Figure 6(a). The temperature of BC-3 rapidly increased to ~100°C in 50 s, while CdS, BiVO_4,_ and TiO_2_ can only reach 43, 43, and 31°C, respectively. With further being irradiated for 240 s, the temperature of BC-3 stabilizes at approximately 113.5°C, CdS, and BiVO_4_ stabilize at 46.2°C, and TiO_2_ stabilizes at 32.3°C, respectively (See Figure S18 for more details). The above experimental results show that the photothermal conversion ability of BC-3 is much better than that of CdS and BiVO_4_, which is caused by the exothermic recombination of photoelectrons from BiVO_4_ and photoholes from CdS. In addition, the good photothermal effect of BC-3 is also supported by the nonradiative transitions of a large number of electrons in BC-3 observing by transient fluorescence measurements. In order to confirm the influence of photothermal effect on photocatalysis, the temperature-dependent photocatalytic experiments were performed. It can be seen from Figure 6(b) that the change of temperature has little effect on the photocatalytic performance of CdS, BiVO_4,_ and TiO_2_. However, for BC-3 (Figure 6(c)), the increase in temperature decreases the time of the slow reaction stage. It is known that water has better thermal conductivity than air, which results in a long time for the local temperature of the catalyst in solution to a temperature that significantly accelerates the rate of catalysis. As the initial temperature of the reaction system increases, the heat loss decreases and the required temperature rise decreases, so the slow reaction phase is gradually shortened. Therefore, it is believed that the photothermal effect accelerates the photocatalytic process.

Based on all the above characterization and experimental results, the photocatalytic extraction mechanism of U(VI) by BC-3 can be further confirmed and supplemented. As shown in Figure 6(d), the unique energy level structure of CdS and BiVO_4_ makes them form an S-scheme heterojunction after compounding, which avoids photocorrosion of CdS while giving BC-3 the strong reduction ability of CdS and the strong oxidation ability of BiVO_4_. In this way, the reduction reaction (the aforementioned Eq. (2), (3), and (4)) can proceed smoothly thanks to the sufficiently negative CBM potential of CdS. At the same time, the strong oxidation ability of BiVO_4_ drives the oxidation reaction (the aforementioned Eq. (5) and (6)) due to the sufficiently positive VBM potential of BiVO_4_. This leads to the result that there is U(IV) in the intermediate products but all U(VI) (UO_2_(OH)_2_) in the final product. In particular, the electrons from BiVO_4_ and holes from CdS in the S-scheme heterojunction undergo a rapid exothermic complexation driven by the internal electric field, band edge bending, and Coulomb interaction, which elevates the local reaction temperature and leads to an accelerated photocatalytic rate.

### 2.3. Influence of External Environment on Photocatalysis

As we know, the amount of catalyst plays a decisive role in the catalytic reaction rate. Therefore, experiments with different amounts of photocatalyst are carried out. It can be seen from Figure S19 that when the initial photocatalyst amount is 10 mg, the reaction rate is very slow, which is unfavorable for the investigation. On the contrary, when the initial photocatalyst amount is 30 mg, the reaction rate is too fast to explore the catalytic process. When the photocatalyst was added at 20 mg (0.4 g/L), it exhibited a suitable reaction rate to study the reaction mechanism, which also happens to be the concentration used in this work.

Figure S20A shows the photocatalysis of U(VI) by BC-3 at different pH values (The corresponding first-order kinetic fitting curves and k values are presented in Figure S21 and S22). At pH = 2.0 and pH = 4.0, the concentration of U(VI) is nearly unchanged, indicating that no photocatalysis occurred. At pH > 6.0, U(VI) is almost completely removed in the dark stage. To find out the reason, the existing forms of U(VI) under different pH values are calculated by Visual MINTEQ 3.1 (Figure S23). The zero potential point of BC-3 is around pH = 2.5 (Figure S2), and the dominant U(VI) species is UO_2_^2+^. Therefore, U(VI) is hard to adsorb on the surface of BC-3 at pH 2.0 because of the electrostatic repulsion effect between the positively charged surface of BC-3 and UO_2_^2+^. When pH > 2.5, the surface of the photocatalyst is negatively charged, which may lead to the weak interaction between U(VI) and BC-3, and a large number of H_3_O^+^ in the solution may compete with the adsorption of UO_2_^2+^ on the surface of BC-3. [56] When pH > 6.0, the U(VI) at a concentration of 70 ppm may self-precipitate to be (UO_2_)_6_O_2_(OH)_8_·6H_2_O, which explains the phenomenon that the residual U(VI) concentration is too low in the dark (Figure S20A). At pH 5.0, BC-3 shows excellent photocatalytic performance. Therefore, a pH value of 5.0 is selected in the follow-up experiments. The effect of foreign ions on photocatalysis is also investigated and shown in Figure S20B and C. It is found that the presence of CO_3_^2-^ may completely impair the photocatalytic performance of BC-3 due to the formation of numerous soluble and stable complexes ((UO_2_CO_3_, UO_2_(CO_3_)_2_^2-^, UO_2_(CO_3_)_3_^4-^, etc). [57] The inhibition of SO_4_^2-^ on photocatalysis may be due to the formation of Bi_2_(SO_4_)_3_ precipitation on the surface of BC-3. [58] However, for Cl^−^_,_ CH_3_COO^−^ (AcO^−^), and NO_3_^−^, they indicate the promotion of the photocatalysis of U(VI). Cl^−^ can capture photoholes to form Cl^∙^, which reduces the recombination rate of photogenerated electron-hole pairs and promotes the reaction. [59] The hydrolysis of AcO^−^ increases the pH value of the system to a certain extent, making the BC-3 surface more negative and resulting in promoted adsorption and photocatalytic performance. As for the cations, only Na^+^ has an obvious accelerating effect on the photocatalysis of U(VI), while the rest have a certain inhibitory effect and could be gradually strengthened with the increase of cationic valence and ion radius. This phenomenon may be caused by the competitive adsorption effect of positively charged ions with UO_2_^2+^ on the BC-3 surface.

The effect of the light wavelength on the photocatalysis of U(VI) is also being investigated. According to the monochromatic photocatalytic experiments (Figure S20D), the best performance of the photoreduction of U(VI) is achieved under the irradiation of 460 nm wavelength light. When the light wavelength is longer than 590 nm, no clear change of the U(VI) concentration is observed as light is on, and a very weak decrease in the U(VI) concentration could be observed under 520 nm light irradiation. However, although BC-3 has good light absorption at 365 nm and 405 nm, it does not have a good photocatalytic effect on U(VI) photoreduction under these light irradiations, which could be because photoelectrons are not completely allowed to be generated under these conditions.

### 2.4. Recycling Performance

As an important performance indicator of catalysts, the recyclability of BC-3 is tested in this work. In the test, the conditions of each experiment are the same as in the first photocatalytic experiment. After each photocatalytic experiment, the BC-3 is washed with 0.1 M Na_2_CO_3_ to elute uranium. As shown in Figure S24, the removal of U(VI) by BC-3 decayed from 85% to 78% after five cycles, which is satisfactory.

## 3. Conclusions

In conclusion, an S-scheme heterojunction CdS/BiVO_4_ photocatalyst is successfully synthesized and used to photothermal-assisted photocatalyze U(VI) efficiently. Systematic analysis and studies have shown the construction of S-scheme heterojunctions, which is confirmed by characterizations such as transient absorption spectroscopy, Tafel curves, etc. The photocatalysis of U(VI) is divided into two processes: reduction by electrons/^•^O_2_^−^ to U(IV) and subsequent reoxidation by holes/^•^OH to insoluble UO_2_(OH)_2_. This extraction process makes full use of photoelectrons and photoholes without scavengers, which is a major inspiration for the development of catalysts with lower cost and wider conditions. In addition, the photoelectrons in the CB of BiVO_4_ are found to transfer to the VB of CdS by releasing a mass of heat which accelerate the photocatalytic rate greatly, allowing BC-3 to maintain the maximum reduction and oxidation capacity and avoiding photocorrosion of CdS. This work discloses for the first time the photocatalytic extraction of U(VI) enhanced by the photothermal effect, widening the way for the development of new photocatalytic materials and strategies.

## 4. Materials and Methods

### 4.1. Reagents and Synthesis

#### 4.1.1. Reagents

All chemicals used in this work, including TiO_2_ (P25) used in the comparative experiments, are purchased from Macklin Biochemical Co. Ltd. (Shanghai, China) and are of analytical purity without any further purification.

#### 4.1.2. Preparation of BiVO_4_

In this experiment, a hydrothermal method was used to prepare BiVO_4_: 0.02 mol of Bi (NO_3_)_3_·5H_2_O and 0.02 mol of NH_4_VO_3_ were dissolved in 20 mL of 65% HNO_3_ and 20 mL of 6 mol/L NaOH solution, respectively. The above two solutions were mixed drop by drop under magnetic stirring for 0.5 h and then continued stirring for 1 h to get a stable and uniform yellow solution. After adjusting the pH of the solution to 7 using dilute NaOH and HCl solutions, the solution was sealed in a 50 mL high-pressure reaction kettle lined with polytetrafluoroethylene (PTFE) and heated to 180°C for 6 h in a blast drying oven. Then, the reactor was naturally cooled to room temperature, and yellow precipitation was obtained after filtration and washed with ethanol and ultrapure water several times. After being dried in a freezing drying oven for 12 h, a yellow BiVO_4_ powder was finally obtained.

#### 4.1.3. Preparation of CdS

CdS was prepared by a simple precipitation method: 0.02 mol of CdCl_2_·2.5H_2_O and 0.02 mol of Na_2_S were dissolved in 20 mL of ultrapure water, respectively. After magnetic stirring for 0.5 h, the latter was poured into the former and stirred for another 2 h. The resulting orange suspension was centrifuged and the precipitation was retained. The orange-red precipitates were washed with ultrapure water and ethanol several times, respectively, then placed in a freezing drying oven for 12 h. Finally, the material is taken out and ground into powder.

#### 4.1.4. The Synthesis of CdS/BiVO_4_ Composite

0.1 mmol of BiVO_4_ was dispersed into 50 mL of ultrapure water, and then a certain amount (0.5 mmol, 0.3 mmol, 0.1 mmol, and 0.03 mmol) of CdCl_2_·2.5H_2_O solid powder was added, and stirred for 1 h, followed by adding the corresponding amount of 1 M of Na_2_S solution (n_Cd_: n_S_ =1: 1). After continuous stirring for 2 h, the liquid in the precipitation was washed 3 times with ethanol and ultrapure water, respectively. Then the product was dried in a freezing drying oven for 12 h. Finally, the sample was ground, and orange composite products were obtained, which were named BC-n (BC-1 (n_BiVO4_: n_CdS_ =3: 1), BC-2 (n_BiVO4_: n_CdS_ =1: 1), BC-3 (n_BiVO4_: n_CdS_ =1: 3), and BC-4 (n_BiVO4_: n_CdS_ =1: 5)).

### 4.2. Characterization Methods

The XRD patterns were gotten by SmartLab SE (Rigaku Corporation). Thermogravimetry (TG) analysis was investigated by the Jupiter thermal analyzer (NETZSCH STA 2500). Zetasizer Nano ZSE and Micro metrics TriStarII 3020 were used to measure zeta potentials and specific surface area, respectively. Energy-dispersive X-ray spectroscopy (EDS) and surface morphology were performed on a field-emission scanning electron microscope (FESEM, Hitachi, SU8010). The transmission electron microscope (TEM), high-resolution TEM (HRTEM), and selected area electron diffraction (SAED) images were obtained from a JEOL JEM 2100 instrument. The absorption spectra and UV-Vis diffuse reflectance spectra (UV-Vis DRS) were measured by UV-2700 (Shimadzu, UV-2700). Fourier-transform infrared (FTIR) transmission spectra were recorded by SHIMADZU-IRT racer-100 with KBr as pellet support. The Cd ions concentration was tested with inductively coupled plasma mass spectrometry (ICP-MS, Agilent Technologies, USA). X-ray photoelectron spectroscopy (XPS, Thermo Fischer, ESCALAB 250Xi, USA, Al K_*α*_ (h*ν* = 1486.7 eV)) was used to characterize the surface chemical components and oxidation states. Fluorescence spectra were recorded by using Perkin Elmer LS 55 Fluorometer. The infrared photothermal images are taken by portable infrared thermography (Hikmicro, P10B) connected to a mobile phone. The time-resolved fluorescence spectra were recorded with an Edinburgh fluorescence spectrometer (FLS980). Information about transient absorption (TA) spectroscopy and surface photovoltage tests are detailed in the Supporting Information (SI).

### 4.3. Photocatalysis Experiments

For photocatalysis experiments, 20 mg photocatalysts are added into 50 mL UO_2_^2+^ solution (70 ppm, obtained by dissolving UO_2_ (NO_3_)_2_·6H_2_O) in a quartz tube. The negligible volume of HCl or NaOH solution is used to adjust the pH value. A 350 W xenon lamp fitted with a 420 nm cut-off filter was used as the light source. Before turning on the light, the solution was subjected to a dark reaction (stirring under dark conditions) for 120 min to reach ad-desorption equilibrium. The concentration of U(VI) in the filtrate is measured by UV-vis spectrophotometry at 650 nm using Arsenazo III as the chromogenic agent. The reagent added in the ion effect experiment is sodium salt (anion) or chloride salt (cation) and the corresponding concentrations are 0.1 M.

### 4.4. Quenching and Free Radical Detection Experiments

In the quenching experiments with the trapping agent concentration of 0.01 M, isopropyl alcohol (IPA) and dimethyl sulfoxide (DMSO) were used as ^•^OH trapping agents. Methanol (Me), ferric chloride (FC), and p-benzoquinone (P-BQ) are used to trap holes, electrons, and ^•^O_2_^−^, respectively. The detection of ^•^OH concentration is based on the method provided in the previous work. [60] In detail, for the detection of ^•^OH, except that the UO_2_^2+^ solution was changed to a 1 mM terephthalic acid solution, the rest of the conditions were the same as in the photocatalytic experiment.

### 4.5. Electrochemical Experiments

The electrochemical workstation model CHI660e (Shanghai Chenhua Instrument Co., Ltd) is used for the electrochemical experiments in this work. In the transient photocurrent test (V_bias_ = 0 V), Tafel tests (scan rate = 0.01 V/s), and Mott-Schottky (MS) plot recording (*f* = 1000 Hz) the electrolyte was 0.2 M, Na_2_SO_4_ solution is used as electrolyte and carbon electrode (GCE), Ag/AgCl electrode and Pt sheet are selected as reference electrode and counter electrode, respectively. A 500 W Xe lamp equipped with a 420 nm cut-off filter is used as the light source in the transient photocurrent test. The electrochemical impedance spectra (EIS) test was recorded at a frequency range of 0.1 Hz - 100 kHz with an amplitude of 5 mV.

### 4.6. Electron Spin Resonance (ESR) Spectroscopy

Brucker A300 ESR spectrometer was used to characterize the formation of ^•^O_2_^−^ and ^•^OH at room temperature. The instrument parameters are set as follows: frequency 9.853 GHz, microwave power 10.8 mW, modulation amplitude 1G, sweep range 3460-3560 G, time constant 1.250 ms, sweep time 19.456 s. 5,5-dimethyl-1-pyrroline N-oxide (DMPO) at a concentration of 50 mM was used to verify the ESR signal of spin-trapping paramagnetic species. Methanol was used as a dispersant for the identification of ^•^O_2_^−^, while ultrapure water was used for ^•^OH.

## Data Availability

All data required to support this study are presented in the paper and the supplementary materials. Additional data are available from the authors upon reasonable request.

## Additional Points

*Highlights:* (1) An S-scheme heterojunction CdS/BiVO_4_ was designed for the photoextraction of U(VI). (2) The S-scheme heterojunction maintains the high redox potentials over the traditional type II charges transfer paths. (3) The photocatalytic reaction was accelerated by prominent photothermal effect. (4) U(VI) extraction was found to involve both reduction and oxidation processes. (5) The photo corrosion of CdS/BiVO_4_ heterojunction and sacrificial agents in the photoextraction of U(VI) are avoided

## Supplementary Materials

Supplementary Materials Experimental section: transient absorption (TA) spectra and Surface photovoltage (SPV) spectra of samples. Discussion on photocurrent test results. Fitting method of time-resolved fluorescence spectroscopy. Table S1: Fitting parameters obtained from the decay curves in TRPL spectra. Figure S1: Adsorption capacity of different samples on U(VI), (Δ*C* = *C*_t_ − *C*_0_). Figure S2: Zeta potential of BC-3 at different pH. Figure S3: First-order kinetic fitting curve. Figure S4: TG curves (in N_2_ atmosphere) of the as-synthesized materials. Figure S5: The nitrogen adsorption-desorption isotherms and the pore size distribution (the inset) of BC-3. Figure S6: FESEM image of CdS. Figure S7: FESEM image of BiVO_4_. Figure S8: FESEM image of BC-3 (A); TEM image of BC-3 (SAED pattern inset) (B); HRTEM image of BC-3 (corresponding to the red boxed part of Figure 2(b)) (C); The FESEM image of BC-3 (D) and EDS mappings of O (E), V (F), Bi (G), S (H), Cd (I). Figure S9: FESEM images of Half-used BC-3 at different resolutions (A and B) and Used-BC-3 (C). Figure S10: Elemental distribution of all elements in Used BC-3. Figure S11: High resolution of XPS spectra of Bi and S (A), O (B), Cd (C), V (D) in Half-used BC-3. Figure S12: High resolution of XPS spectra of Bi and S (A), O (B), Cd (C), V (D) in Used BC-3. Figure S13: High-resolution XPS spectrum of U 4f in Used BC-3 of the repeated experiment. Figure S14: Fluorescence spectra of the terephthalic acid solution under BC-3 with irradiation (*λ*_ex_ = 310 nm) (A) and the corresponding concentration of ^•^OH (B). Figure S15: The schematic diagrams for the energy band configuration of Type II (A) and Z-scheme (B) for BC-3. Figure S16: EIS spectra of different samples in 0.1 M Na_2_SO_4_ solution at a potential of 0.1 V. Figure S17 Fluorescence spectra of the as-synthesized samples. Figure S18: The temperature changes of BC-3, CdS, BiVO_4,_ and TiO_2_ with time under the irradiation of xenon lamp (350 W, >420 nm). Figure S19: Effect of input amount of BC-3 on photocatalytic activity. Figure S20: Effects of pH (A), anions (B), and cations (C) on photocatalysis of U(VI) by BC-3. The photocatalytic performance of BC-3 under light irradiation with different wavelengths (D). Figure S21: The corresponding first-order kinetic fitting curves in Figure S20. Figure S22: the corresponding k value of first-order kinetic fitting curves in Figure S20. Figure S23: The pH-dependent speciation of U(VI) in solution. *C*_U(VI) initial_ = 70 mg/L, *T* = 298 K. Figure 24: Recyclability test of BC-3 for U(VI) photocatalysis. Reaction conditions: 20 mg BC-3 are suspended in 50 mL of 70 mg/L UO_2_^2+^ aqueous solution.
