# Supplementary material for: Insights into Photothermally Enhanced Photocatalytic U(VI) Extraction by a Step-Scheme Heterojunction
Source: Research (Wash D C). 2022 Oct 11;2022:9790320. doi: 10.34133/2022/9790320 (PMC9590271; doi:10.34133/2022/9790320)
Supplement: Supplementary Materials — Experimental section: transient absorption (TA) spectra and Surface photovoltage (SPV) spectra of samples. Discussion on photocurrent test results. Fitting method of time-resolved fluorescence spectroscopy. Table S1: Fitting parameters obtained from the decay curves in TRPL spectra. Figure S1: Adsorption capacity of different samples on U(VI), (ΔC = Ct − C0). Figure S2: Zeta potential of BC-3 at different pH. Figure S3: First-order kinetic fitting curve. Figure S4: TG curves (in N2 atmosphere) of the as-synthesized materials. Figure S5: The nitrogen adsorption-desorption isotherms and the pore size distribution (the inset) of BC-3. Figure S6: FESEM image of CdS. Figure S7: FESEM image of BiVO4. Figure S8: FESEM image of BC-3 (A); TEM image of BC-3 (SAED pattern inset) (B); HRTEM image of BC-3 (corresponding to the red boxed part of Figure 2(b)) (C); The FESEM image of BC-3 (D) and EDS mappings of O (E), V (F), Bi (G), S (H), Cd (I). Figure S9: FESEM images of Half-used BC-3 at different resolutions (A and B) and Used-BC-3 (C). Figure S10: Elemental distribution of all elements in Used BC-3. Figure S11: High resolution of XPS spectra of Bi and S (A), O (B), Cd (C), V (D) in Half-used BC-3. Figure S12: High resolution of XPS spectra of Bi and S (A), O (B), Cd (C), V (D) in Used BC-3. Figure S13: High-resolution XPS spectrum of U 4f in Used BC-3 of the repeated experiment. Figure S14: Fluorescence spectra of the terephthalic acid solution under BC-3 with irradiation (λex = 310 nm) (A) and the corresponding concentration of •OH (B). Figure S15: The schematic diagrams for the energy band configuration of Type II (A) and Z-scheme (B) for BC-3. Figure S16: EIS spectra of different samples in 0.1 M Na2SO4 solution at a potential of 0.1 V. Figure S17 Fluorescence spectra of the as-synthesized samples. Figure S18: The temperature changes of BC-3, CdS, BiVO4, and TiO2 with time under the irradiation of xenon lamp (350 W, >420 nm). Figure S19: Effect of input amount of BC-3 on [file 9790320.f1.docx]

Supporting Information

**Insights into photothermally enhanced photocatalytic U(VI) extraction by a step-scheme heterojunction**

Yifeng Zhang,^a⸸^ Haorong Sun,^a⸸^ Feixue Gao,^a^ Shuo Zhang,^a^ Qingzhi Han,^b^ Jing Li,^c^ Ming Fang,^a*^ Yawen Cai,^d^ Baowei Hu,^d^ Xiaoli Tan,^d*^ Xiangke Wang,^a*^

^a^ MOE Key Laboratory of Resources and Environmental System Optimization, College of Environmental Science and Engineering, North China Electric Power University, Beijing, 102206, PR China

^b^ Key Laboratory of Theoretical and Computational Photochemistry, Ministry of Education, College of Chemistry, Beijing Normal University, Beijing, 100875, PR China

^c^ Key Laboratory of Photochemical Conversion and Optoelectronic Materials, Technical Institute of Physics and Chemistry, Chinese Academy of Sciences, Beijing 100190, China

^d^ School of Life Science, Shaoxing University, Shaoxing 312000, P.R. China

⸸ The same contribution

Author to whom should be corresponded: Ming Fang (mfang@ncepu.edu.cn), Xiaoli Tan (xltan@ncepu.edu.cn), Xiangke Wang (xkwang@ncepu.edu.cn)

**Experimental section**

**Testing of transient absorption (TA) spectra**

The regenerative-amplified Ti: sapphire laser (Coherent) and Helios pump-probe system (Ultrafast Systems) were used for transient absorption (TA) spectroscopy measurements. The output light (Vitara/Legend Elite-1K-HE, producing 25-fs pulses at 1 kHz with pulse energies of 4 mJ near 800 nm) was split into two parts to generate the pump and probe pulses. The main part was directed into the optical parametric amplifiers (TOPAS-C), which generated a 320 nm laser pulse for the pump beam. A small part of the output light passed through the motorized optical delay line and was focused on a sapphire crystal to generate the white-light continuum probe pulses with a wavelength of 450 to 820 nm, which was used for the probe light. For the pump, chopped by a synchronized chopper at a frequency of 500 Hz, the pump pulses were modulated such that the TA spectra with and without the pump pulses could be recorded alternately. The intensity of the pump pulse used in the experiment was controlled by a variable neutral-density filter wheel. For the probe, the WLC beam was focused with a parabolic reflector onto the sample. After sampling, the probe beam was collimated and then focused into a fiber-coupled spectrometer and detected at a frequency of 1 kHz. The delay time between the pump light and the probe light was controlled using a motorized optical delay-line. All experiments were performed at room temperature. The time profiles of TA spectra are fitted with the following bi-exponential function.

*I*_(_*_t_*_)_ = *I*_(0)_ + *A*_1_exp(-*t*/*τ*_1_) + *A*_2_exp(-*t*/*τ*_2_)

**Surface photovoltage (SPV) spectra**

The SPV measurements were carried out with a home-built apparatus including a 500 W xenon lamp (CHF XM500W, Beijing Trusttech Co. Ltd), a double-grating monochromator (Zolix SP500), a lock-in amplifier (SR830-DSP) with an optical chopper (SR540) and a sample cell.

**Discussion on photocurrent test results**

In electrochemistry, anode photocurrent means that an oxidation reaction takes place on the working electrode, while cathode photocurrent means that a reduction reaction takes place on the working electrode.[1] In the transient photocurrent test (Figure 3E), CdS shows anode photocurrent while BiVO_4_ shows cathode photocurrent, which means that the photoholes in CdS and the photoelectrons in BiVO_4_ dominate the photocurrents in the UO_2_^2+^ solution. Or in other words, the CdS acts as an electron acceptor, and BiVO_4_ acts as an electron donor under light irradiation. Therefore, as CdS is combined with BiVO_4_, the photoelectrons in BiVO_4_ are likely to transfer to CdS.

**Time-resolved fluorescence spectroscopy**

The results of time-resolved fluorescence spectroscopy are fitted with the following biexponential function [2, 3]:

*I*_(_*_t_*_)_ = *I*_(0)_ + *A*_1_exp(-*t*/*τ*_1_) + *A*_2_exp(-*t*/*τ*_2_)

Where *I*_0_ represents the baseline correction value, *τ* denotes the lifetime of the different energy transfer processes (*τ*_1_ and *τ*_2_ refer to radiative and non-radiative, respectively) and *A* (*A*_1_, and *A*_2_) are the corresponding pre-exponential factors. The average lifetime (*τ*_ave_) of the as-synthesized samples is calculated according to the following equation and the related results are shown in Table S1:

*τ_ave_*= (*A*_1_τ2 1 + *A*_2_τ2 2)/(*A*_1_τ_1_ + *A*_2_τ_2_)

Table S1 Fitting parameters obtained from the decay curves in TRPL spectra.

| Sample | *τ*_ave_ (ns) | *τ*_1_ (ns) (Rel%) | *τ*_2_ (ns) (Rel%) |
| --- | --- | --- | --- |
| CdS | 2.26 | 0.73 (60.47%) | 4.61 (39.53%) |
| BiVO_4_ | 2.63 | 0.76 (63.60%) | 5.72 (36.40%) |
| BC-3 | 3.00 | 0.87 (59.88%) | 6.36 (40.12%) |





Figure S1: Adsorption capacity of different samples on U(VI), (Δ*C* = *C*_t_-*C*_0_).





Figure S2: Zeta potential of BC-3 at different pH.





Figure S3: First-order kinetic fitting curve.





Figure S4: TG curves (in N_2_ atmosphere) of the as-synthesized materials.





Figure S5: The nitrogen adsorption-desorption isotherms and the pore size distribution (the inset) of BC-3.


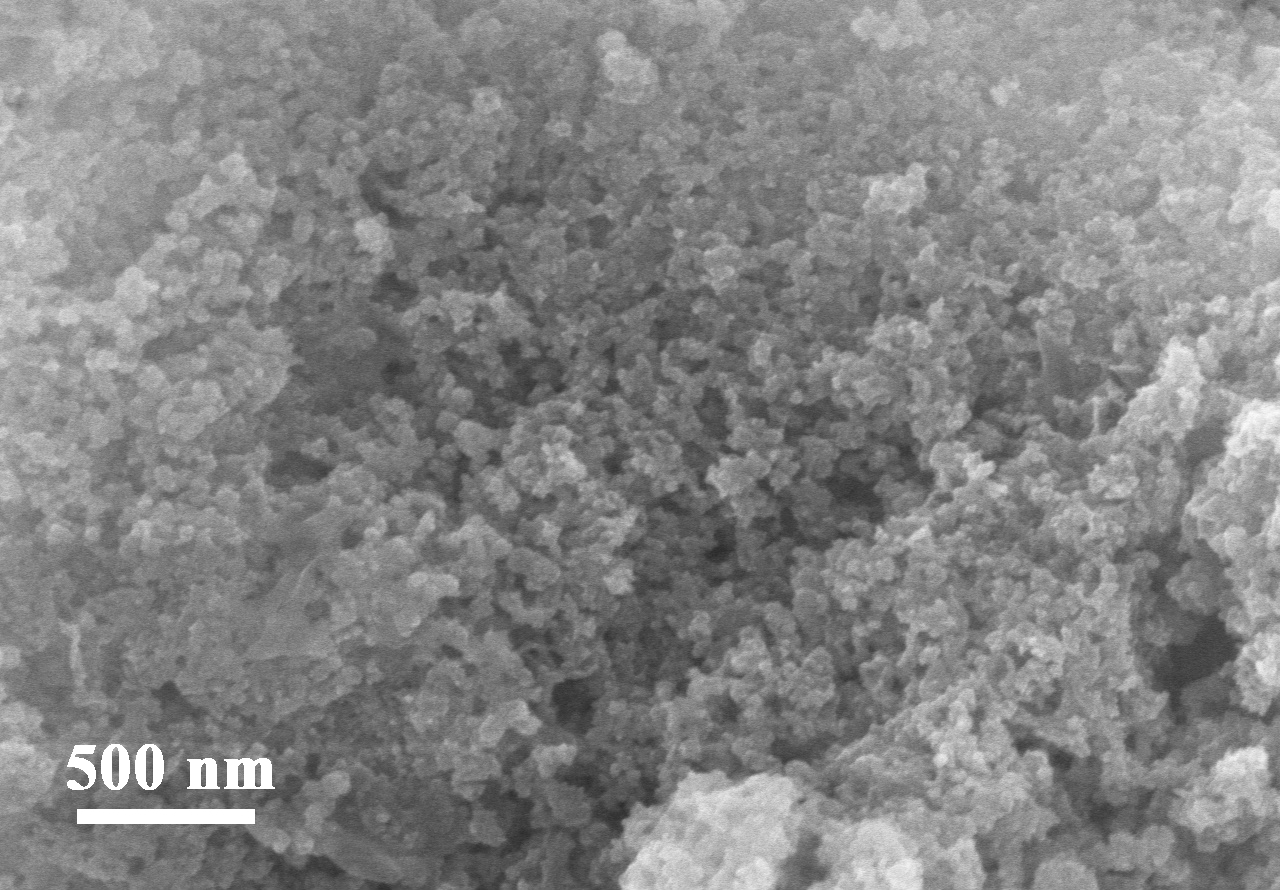


Figure S6: FESEM image of CdS.


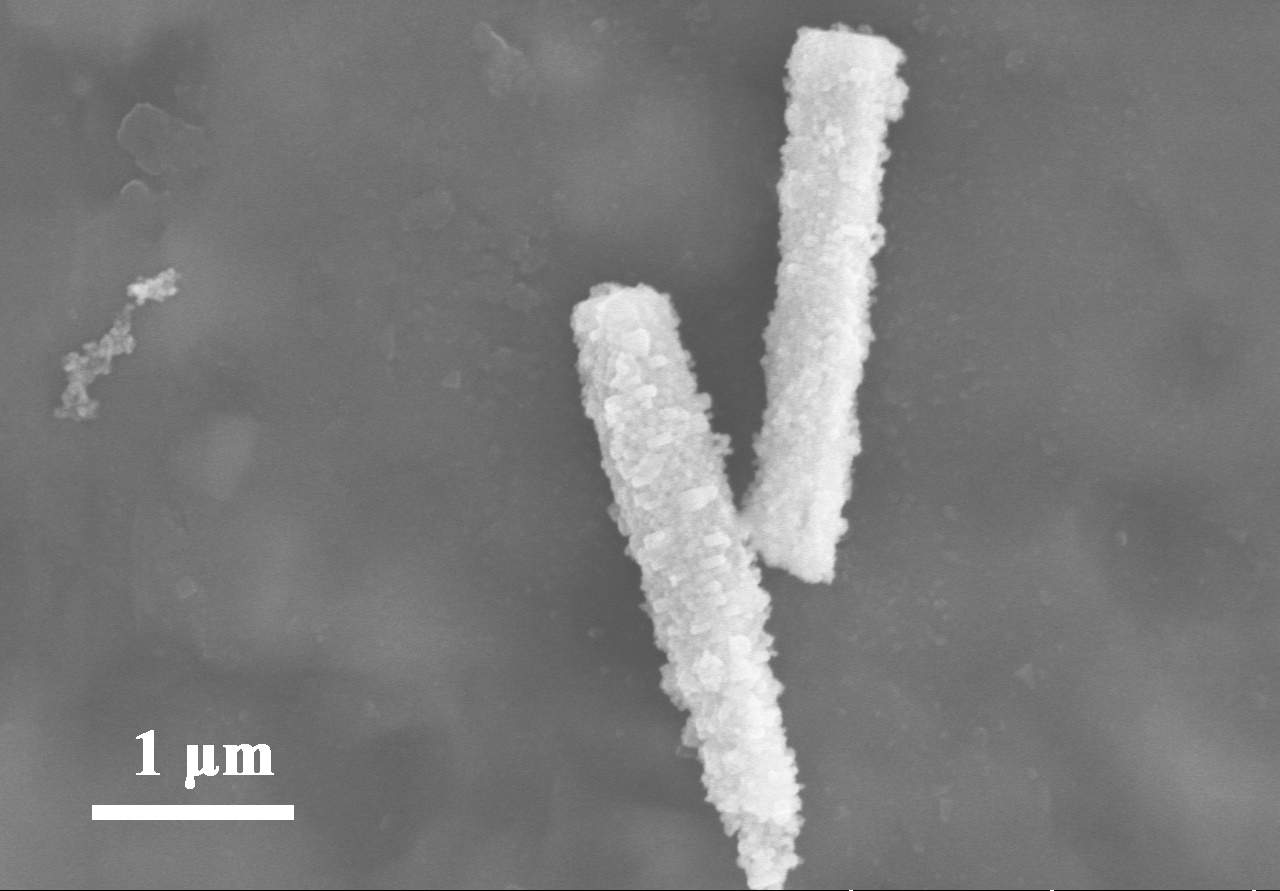


Figure S7: FESEM image of BiVO_4_.


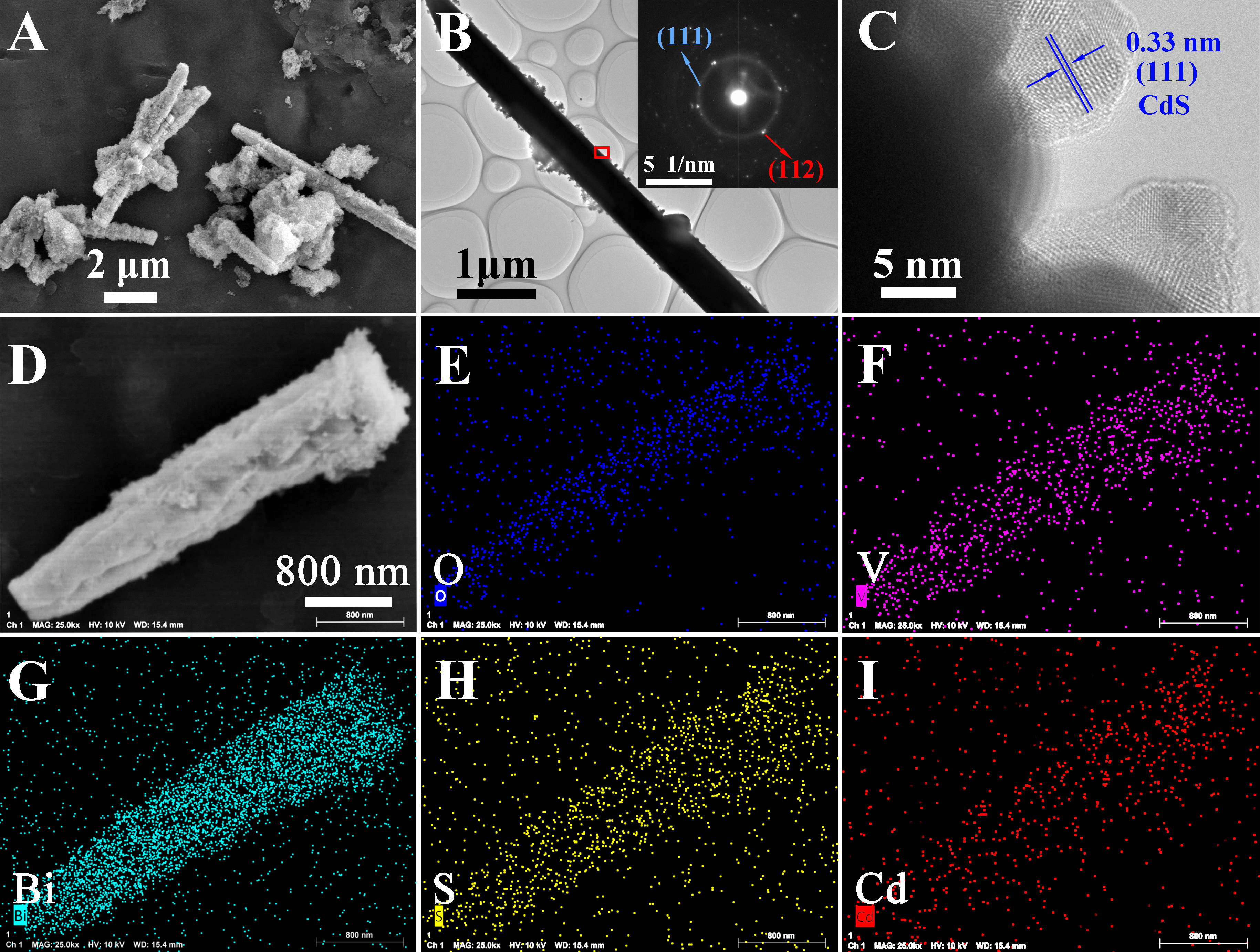


Figure S8: FESEM image of BC-3 (A); TEM image of BC-3 (SAED pattern inset) (B); HRTEM image of BC-3 (corresponding to the red boxed part of Figure 2B) (C); The FESEM image of BC-3 (D) and EDS mappings of O (E), V (F), Bi (G), S (H), Cd (I).


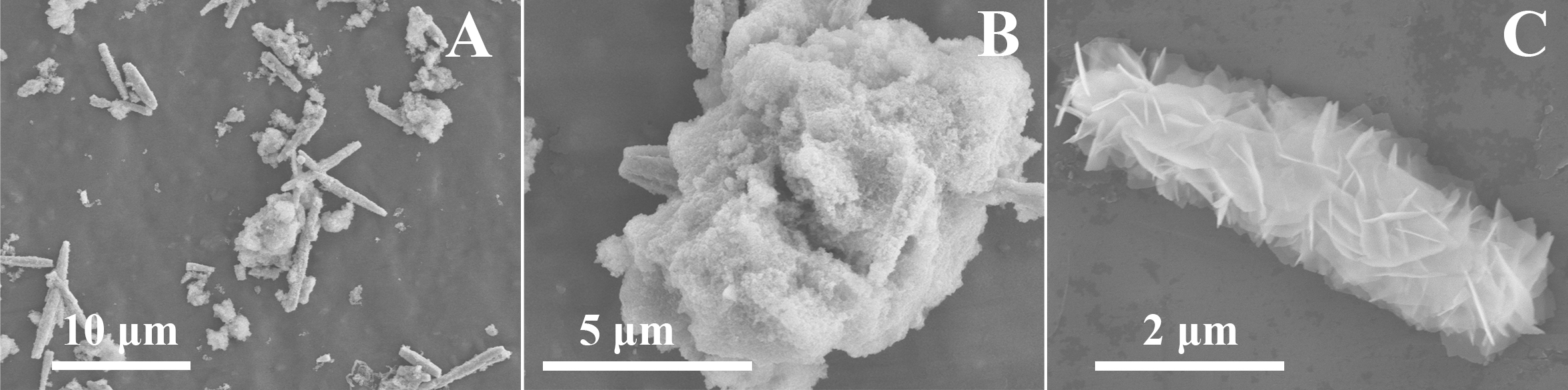


Figure S9: FESEM images of Half-used BC-3 at different resolutions (A and B) and Used-BC-3 (C).


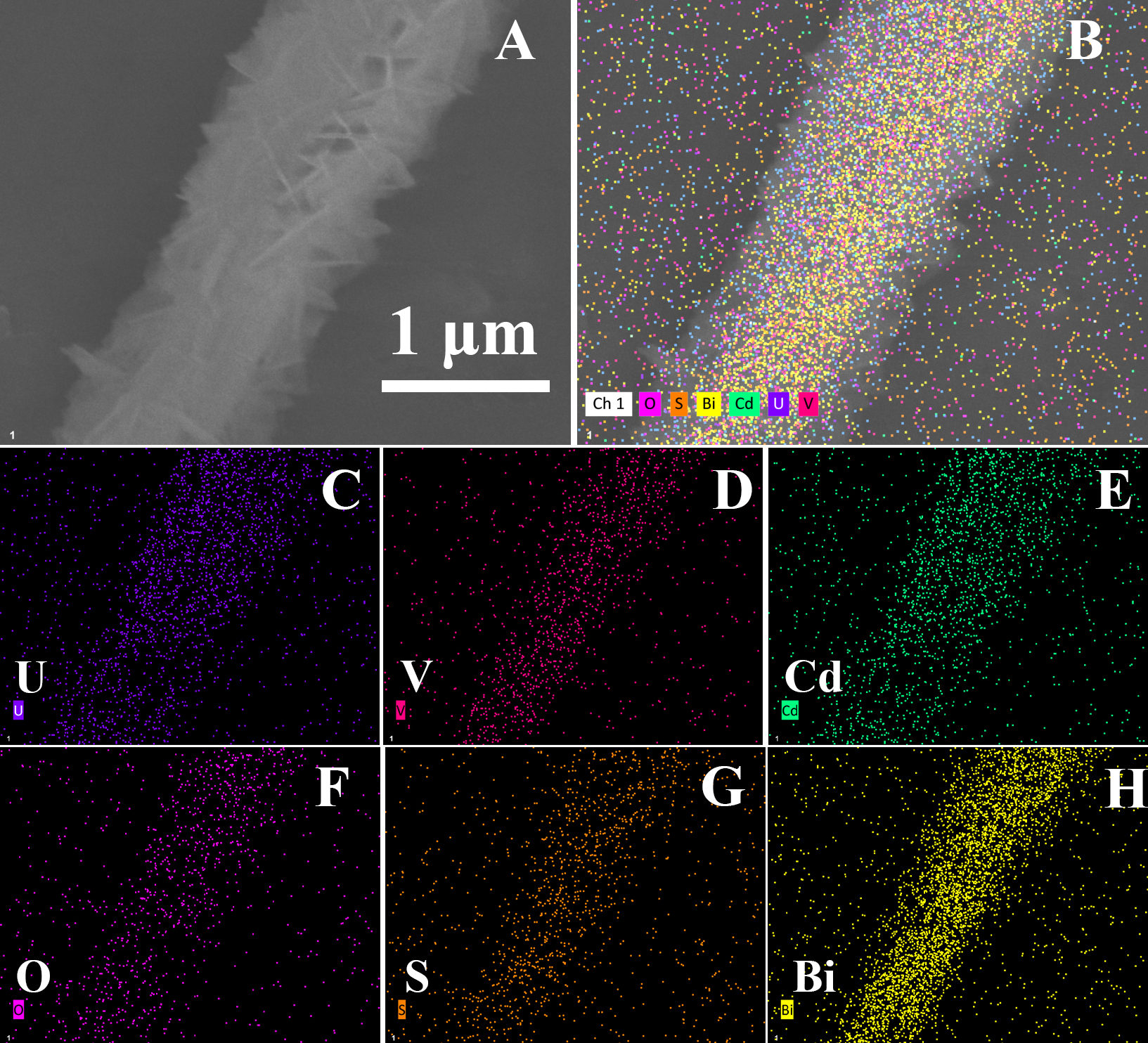


Figure S10: Elemental distribution of all elements in Used BC-3.





Figure S11: High resolution of XPS spectra of Bi and S (A), O (B), Cd (C), V (D) in Half-used BC-3.





Figure S12: High resolution of XPS spectra of Bi and S (A), O (B), Cd (C), V (D) in Used BC-3.





Figure S13: High-resolution XPS spectrum of U 4f in Used BC-3 of the repeated experiment.





Figure S14: Fluorescence spectra of the terephthalic acid solution under BC-3 with irradiation (λ_ex_ = 310 nm) (A) and the corresponding concentration of ^•^OH (B).


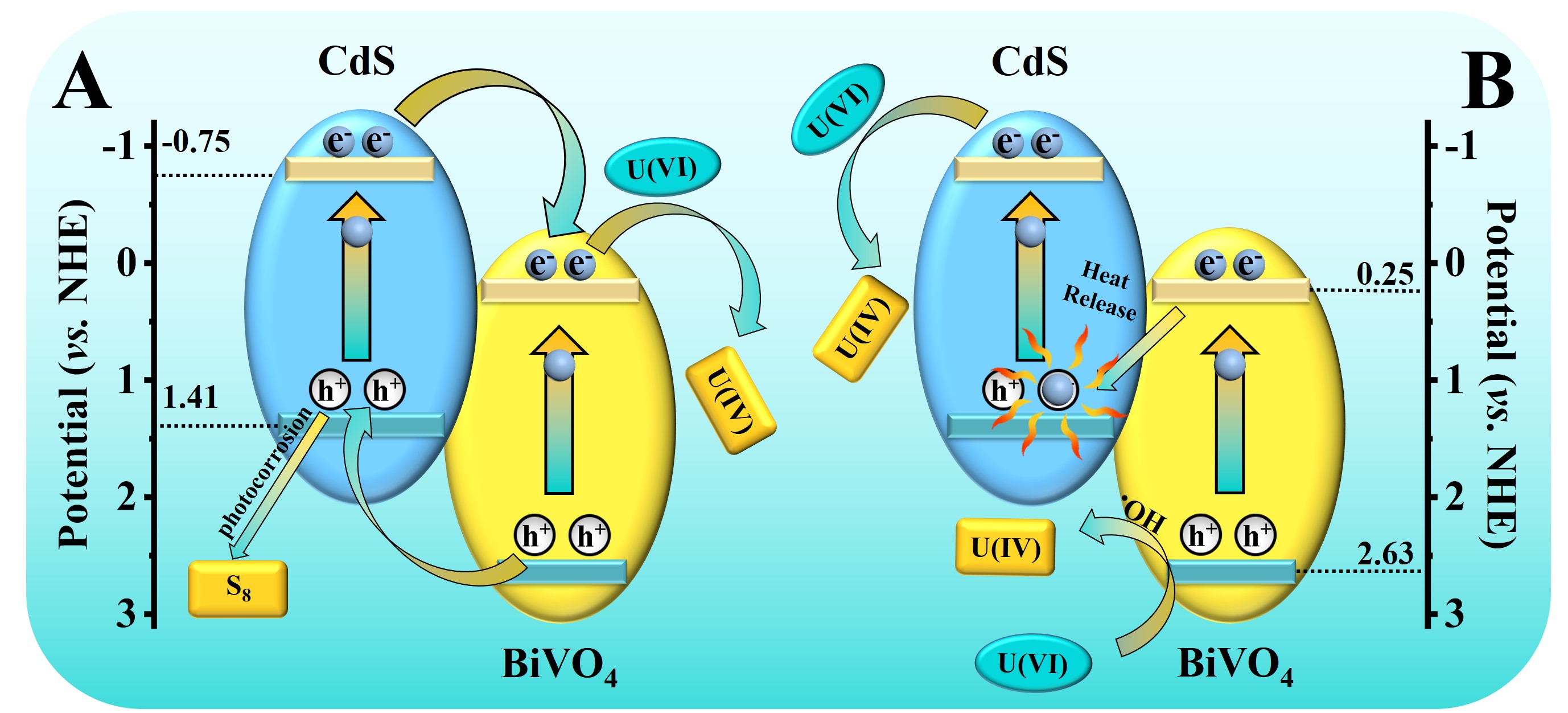


Figure S15: The schematic diagrams for the energy band configuration of Type II (A) and Z-scheme (B) for BC-3.





Figure S16: EIS spectra of different samples in 0.1 M Na_2_SO_4_ solution at a potential of 0.1 V (inset: the equivalent circuit diagram).





Figure S17 Fluorescence spectra of the as-synthesized samples.


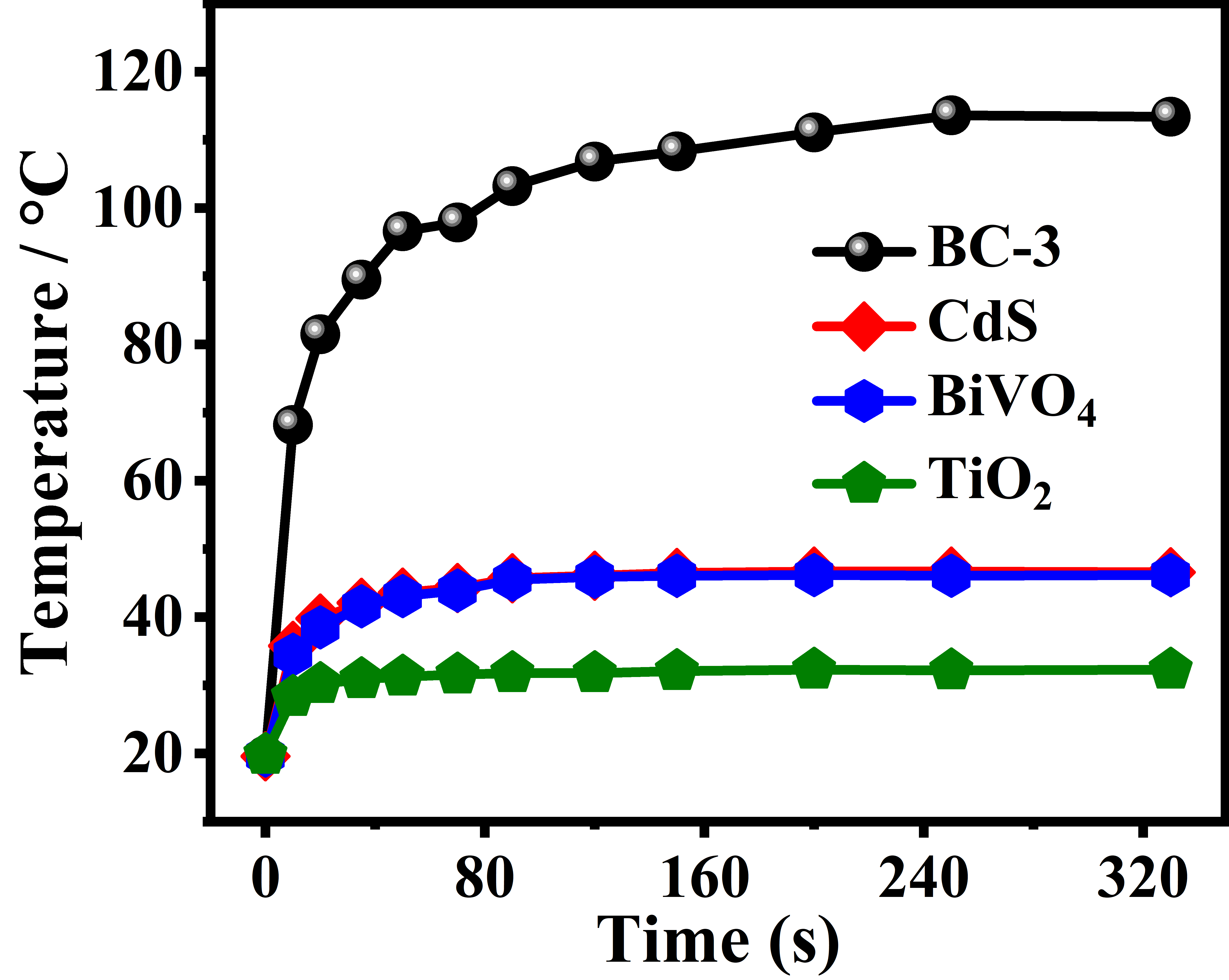


Figure S18: The temperature changes of BC-3, CdS, BiVO_4,_ and TiO_2_ with time under the irradiation of xenon lamp (350 W, > 420 nm).





Figure S19: Effect of input amount of BC-3 on photocatalytic activity.





Figure S20: Effects of pH (A), anions (B), and cations (C) on photocatalysis of U(VI) by BC-3. The photocatalytic performance of BC-3 under light irradiation with different wavelengths (D).





Figure S21: The corresponding first-order kinetic fitting curves in Figure S20.

.





Figure S22: the corresponding k value of first-order kinetic fitting curves in Figure S20.





Figure S23: The pH-dependent speciation of U(VI) in solution. *C*_U(VI) initial_ = 70 mg/L, T = 298 K.


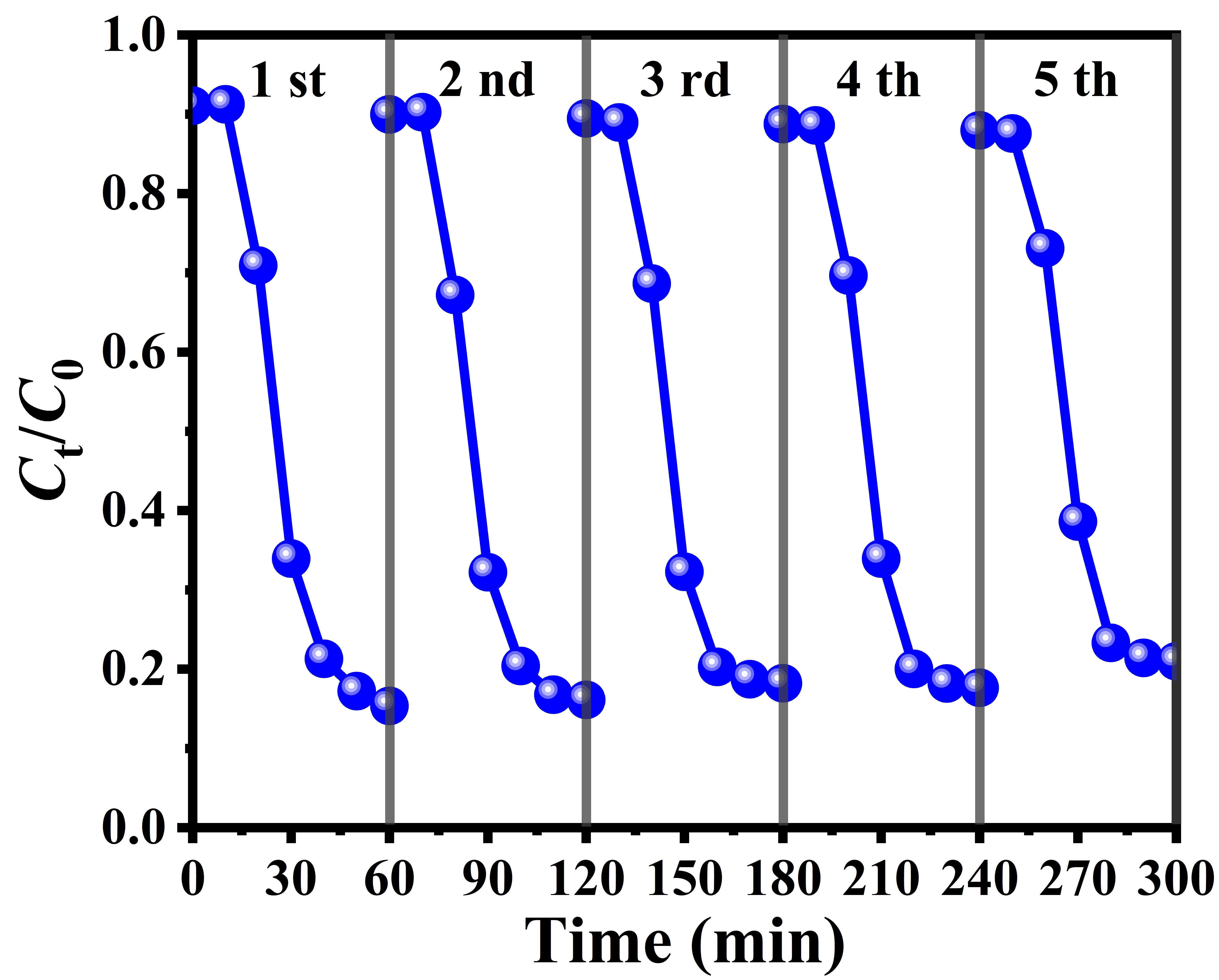


Figure 24: Recyclability test of BC-3 for U(VI) photocatalysis. Reaction conditions: 20 mg BC-3 are suspended in 50 mL of 70 mg/L UO_2_^2+^ aqueous solution.

**References**

[1] D.-D. Qin, Y.-L. Li, T. Wang, et al., “Sn-doped hematite films as photoanodes for efficient photoelectrochemical water oxidation,” *Journal of Materials Chemistry A*, vol. 3, no. 13, pp. 6751-6755, 2015.

[2] C. Cheng, B. He, J. Fan, et al., “An inorganic/organic S‐scheme heterojunction H_2_‐production photocatalyst and its charge transfer mechanism,” *Advanced Materials*, vol. 33, no. 22, pp. 2100317, 2021.

[3] P. Xia, S. Cao, B. Zhu, et al., “Designing a 0D/2D S‐scheme heterojunction over polymeric carbon nitride for visible‐light photocatalytic inactivation of bacteria,” *Angewandte Chemie International Edition*, vol. 59, no. 13, pp. 5218-5225, 2020.
